# Genetic Characterization of Palyam Serogroup Viruses Isolated in Japan from 1984 to 2018 and Development of a Real-Time RT-PCR Assay for Broad Detection of Palyam Serogroup Viruses and Specific Detection of Chuzan (Kasba) and D’Aguilar Viruses

**DOI:** 10.3390/pathogens13070550

**Published:** 2024-06-28

**Authors:** Hiroaki Shirafuji, Natsumi Kishida, Katsunori Murota, Yuto Suda, Tohru Yanase

**Affiliations:** 1 Exotic Disease Group, Division of Transboundary Animal Disease Research, National Institute of Animal Health (NIAH), National Agriculture and Food Research Organization (NARO), 6-20-1 Josuihoncho, Kodaira 187-0022, Tokyo, Japan; 2 Virus Group, Division of Infectious Animal Disease Research, National Institute of Animal Health (NIAH), National Agriculture and Food Research Organization (NARO), 3-1-5 Kannondai, Tsukuba 305-0856, Ibaraki, Japan; natsumi.k@naro.affrc.go.jp (N.K.); suday965@affrc.go.jp (Y.S.); 3 Epidemiology and Arbovirus Group, Division of Transboundary Animal Disease Research, National Institute of Animal Health (NIAH), National Agriculture and Food Research Organization (NARO), 2702 Chuzan, Kagoshima 891-0105, Kagoshima, Japan; k.murota@naro.affrc.go.jp (K.M.); tyanase@affrc.go.jp (T.Y.)

**Keywords:** arbovirus, cattle, real-time RT-PCR, Palyam virus, phylogenetic analysis, serotype

## Abstract

We performed whole genome sequencing (WGS) of 15 Palyam serogroup virus (PALV) strains isolated from cattle or *Culicoides* biting midges in Japan from 1984 to 2018. We found that the PALV strains consisted of Chuzan (Kasba) virus (CHUV), D‘Aguilar virus (DAGV), Bunyip Creek virus, and another PALV, Marrakai virus (MARV). The Japanese MARV strains isolated in 1997 were closely related to Australian PALV strains isolated in 1968–1976 in genome segments 2 and 10, but they were most closely related to other Japanese PALV strains in the other genome segments. Our data suggest that the Japanese MARV strains were reassortant viruses between Asian and Australian PALVs. In addition to the WGS, we developed a real-time reverse-transcription polymerase chain reaction assay that can broadly detect PALV and specifically detect CHUV and DAGV, utilizing the data obtained by the WGS in this study. We detected the DAGV gene in bovine stillborn fetuses and congenitally abnormal calves in 2019 using the newly developed assay. To our knowledge, this is the first report of isolation of MARV outside of Australia and the first report of detection of PALV in bovine fetuses or calves with congenital abnormality outside of Africa.

## 1. Introduction

*Orbivirus palyamense* is a member of the genus *Orbivirus* belonging to the family *Sedoreoviridae*. This virus is commonly described as the Palyam serogroup, and 13 serotypes have been recognized to date: Palyam, Kasba, Vellore, Abadina, D’Aguilar, Nyabira, CSIRO Village, Marrakai, Gweru, Bunyip Creek, Petero, Marondera, and Kindia [1]. The virus is transmitted by arthropod vectors, and some serotypes of this virus are associated with abortion and teratology in cattle and possibly other ruminants [1]. Chuzan virus (CHUV) was originally isolated from bovine blood and *Culicoides oxystoma* in Japan in 1985 and later identified as Kasba virus [2,3]. CHUV causes Chuzan disease in cattle, which is characterized by congenital abnormalities with hydranencephaly-cerebellar hypoplasia (HCH) syndrome in calves and affected calves can cause impairment of mobility and neurological symptoms [4,5,6]. An epidemic of Chuzan disease occurred in Japan between November 1987 and April 1988, affecting at least 2463 calves [5,6]. Additionally, another Palyam serogroup virus (PALV), D’Aguilar virus (DAGV), is thought to be involved in bovine stillbirths and congenital abnormalities in calves [7].

PALV has a 10-segmented, double-stranded RNA (dsRNA) genome encoding at least seven structural proteins (VP1–VP7) and four non-structural proteins (NS1–NS3, NS3a) [8,9,10]. Two of the 10 genome segments, genome segments (Seg-) 2 and 6, encode outer-capsid proteins VP2 and VP5, respectively [11]. VP2 and VP5 are responsible for virus neutralization and serotype specificity, and thus, their amino acid sequences are highly variable [1]. Phylogenetic analysis based on Seg-5, 7 and 9 showed that PALV strains with close geographical origins tend to form the same groups [7,12,13,14]. Additionally, phylogenetic analysis based on the concatenated dataset showed that PALV prototype strains can be divided into two distinct clades, one containing all Afrotropical strains except for Petero virus, the other containing Petero virus and all the strains from the Orient and Australasian regions [11].

PALVs have been isolated in Africa (South Africa, Zimbabwe, and the Central African Republic), Asia (India, Japan, and China), and Australia [1,15,16]. In Japan, the whole genome sequence of the prototype strain of CHUV has been determined [9], but sequence data of other PALV strains are limited to partial sequences, and the relationship between Japanese and foreign PALV strains is not well understood. Therefore, the first objective of this study was to analyze whole genome sequences (WGS) of Japanese PALV strains isolated from cattle or *Culicoides* biting midges in Japan between 1984 and 2018.

A conventional reverse-transcription polymerase chain reaction (RT-PCR) assay has been used to diagnose congenital abnormalities in cattle and identify viruses isolated from sentinel cattle or *Culicoides* biting midges in Japan [17]. However, no PALV has been detected in affected fetuses or calves, likely because most of the PALV in affected fetuses or calves is eliminated during the several months between when the dams are infected with CHUV or DAGV and when abnormal calving occurs. Therefore, diagnosis is usually based on the detection of neutralizing antibodies in serum from calves that have not ingested colostrum, pathological findings of the fetuses or calves, and seroprevalence of sentinel cattle. A problem with the diagnosis is that if an affected calf acquires maternal antibodies by ingesting colostrum, it is not possible to distinguish whether the neutralizing antibodies originate from the dam or are produced by the calf, making it difficult to identify the calf’s infection. To solve this problem, it is necessary to detect the virus, rather than neutralizing antibodies, in the affected calves, and it is thus necessary to develop a highly sensitive assay that can detect PALV, especially CHUV and DAGV. To date, no real-time RT-PCR assay to detect PALV has been reported in a peer-reviewed journal, although some have been developed [18,19]. Since many PALVs have been isolated in Japan, we believe that developing a real-time RT-PCR assay utilizing the sequence data of Japanese PALV strains, as well as PALVs isolated in other countries, will provide higher sensitivity and specificity. Thus, the second objective was to develop a real-time RT-PCR assay that can detect CHUV, DAGV, and other PALVs utilizing the sequence data of Japanese PALV strains to be analyzed in this study.

Overall, this study aims to fill the knowledge gaps in the genetic characterization of Japanese PALV by the WGS and to develop a real-time RT-PCR assay for the detection of PALV with high sensitivity and specificity. The successful completion of these objectives will significantly advance our understanding of PALV epidemiology and enhance our ability to diagnose PALV-related diseases in cattle.

## 2. Materials and Methods

### 2.1. Viruses

The fifteen Japanese PALV strains used for genetic analysis in this study are listed in Table 1. The strains were originally isolated from bovine erythrocytes or *Culicoides* biting midges between 1984 and 2018 in four prefectures in Kyushu Island and Okinawa Prefecture in southwestern Japan (Figure S1). Additionally, two more Japanese PALV strains (KSB-29/E/01 and ON-1/E/08 isolated in 2001–2008), five Australian PALV strains (DPP66, B8112, CSIRO 58, CSIRO 11 and CSIRO 82 isolated in 1968–1981) and a Zimbabwean PALV strain (VRL792/73 isolated in 1973) were used for developing a real-time RT-PCR assay. All the strains were propagated in hamster lung (HmLu-1) cell cultures until approximately 90% of the monolayer showed cytopathic effects (CPE).

### 2.2. Full-Length Amplification of cDNAs (FLAC), Next-Generation Sequencing (NGS) and Sanger Sequencing

For whole genome sequencing (WGS), the 15 Japanese PALV strains were propagated in HmLu-1 cell cultures. We then extracted viral dsRNA from the infected cell cultures and ligated an anchor primer (p-GACCTCTGAGGATTCTAAAC/iSp9/TCCAGTTTAGAATCC-OH) for full-length amplification of cDNAs (FLAC) to the 3′-termini of the dsRNA using T4 RNA ligase 1 (New England BioLabs, Ipswich, MA, USA) as described [20,21]. The viral dsRNA ligated to the anchor primer was purified by using the Monarch RNA Cleanup Kit (New England BioLabs) and then subjected to double-stranded cDNA synthesis by using the NEBNext RNA Ultra First Strand Synthesis Module and the NEBNext RNA Ultra Second Strand Synthesis Module (New England BioLabs). We used the cDNA to prepare libraries for the next-generation sequencing (NGS) process using the TruSeq DNA Nano LT Library Prep Kit (Illumina, San Diego, CA, USA). We then loaded the libraries on an iSeq 100 i1 Reagent (Illumina) and sequenced on iSeq 100 (Illumina). The sequence reads were subjected to de novo assembly by using CLC Genomics Workbench 12 (Qiagen, Hilden, Germany), and the possible viral sequences were identified from contigs to compare sequences in GenBank using BLASTx. We amplified probable gaps between the obtained contigs by RT-PCR and sequenced the products by using BigDye Terminator v 3.1 (Thermo Fisher Scientific, Waltham, MA, USA) on an ABI 3100-Avanti Genetic Analyzer (Thermo Fisher Scientific).

### 2.3. Phylogenetic Analysis

To analyze the phylogenetic relationships among PALV strains, we aligned the coding sequence of each genome segment using CLUSTAL W (version 2.1) [22] and constructed phylogenetic trees with MEGA X software (version 10.2.6) using the method of maximum likelihood [23]; we evaluated the reliability of the branching orders by the bootstrap test (1000 replicates). We used GENETYX version 15 (Genetyx, Tokyo, Japan) to calculate sequence identities among the strains. The nucleotide sequence data reported herein have been deposited in the DNA Data Bank of Japan (DDBJ) under accession numbers LC601654–LC601657, LC601662–LC601669, LC601674–LC601685, and LC818215–LC818340.

### 2.4. Primer and Probe Design for a Real-Time RT-PCR Assay

The sequences of the primers and probes for the real-time RT-PCR assay are shown in Table 2. Two sets of primers and a probe were designed for the broad-range detection of PALV: a set of primers (PALV/S9/182F and PALV/S9/246R) and a probe (PALV/S9/204P) based on conserved regions of PALV Seg-9 (Pan-PALV-1 set), and another set of primers (PALV/S7/1047F and PALV/S7/1109R) and a probe (PALV/S7/1067P) based on conserved regions of PALV Seg-7 (Pan-PALV-2 set). Additionally, two other sets of primers and a probe were designed for specific detection of CHUV or DAGV: a set of primers (CHUV/S2/316F and CHUV/S2/443R) and a probe (CHUV/S2/365P) based on conserved regions of CHUV Seg-2 (Chuzan-specific set), and another set of primers (DAGV/S2/10F and DAGV/S2/99R) and a probe (DAGV/S2/49P) based on conserved regions of DAGV Seg-2 (D’Aguilar-specific set). All the primers and probes were designed using the sequence data obtained by the WGS in this study and other PALV sequence data available in GenBank. The sequences of the primers and probes were prescreened with a free web-based tool for analyzing primers, Net Primer (Premier Biosoft International, Palo Alto, CA, USA), for the real-time RT-PCR assays to work properly. The sequences of the primers and probes were also checked with the Basic Local Alignment Search Tool (BLAST) to prevent non-specific reactions.

### 2.5. A Real-Time RT-PCR Assay

The conditions for a real-time RT-PCR assay were optimized with the SuperScript III Platinum One-Step Quantitative RT-PCR System (Invitrogen/Life Technologies, Carlsbad, CA, USA) and a real-time PCR system, MyiQ_2_ (BIO-RAD, Hercules, CA, USA). For each RNA sample, 2 μL was mixed with 23 μL of reaction mix containing 12.5 μL of 2× Reaction Mix (included in the kit), 10 pmol of each primer, 3 pmol of each probe, 0.5 μL of SuperScript III RT/Platinum *Taq* Mix (kit), 0.5 μL of RNase OUT Recombinant Ribonuclease Inhibitor (Invitrogen) and nuclease-free water. Since the MyiQ_2_ can detect two excited fluorophores, the assays were designed to be multiplex assays to detect 6-carboxyfluorescein (FAM) and 6-VIC or hexachloro-6-carboxyfluorescein (HEX) fluorophores in each tube. The probes of Pan-PALV-1 and Chuzan-specific sets (PALV/S9/204P and CHUV/S2/365P) were both labeled with FAM at the 5′ end, the probe of Pan-PALV-2 set (PALV/S7/1067P) was labeled with VIC at the 5′ end, and the probe of D’Aguilar-specific set (DAGV/S2/49P) was labeled with HEX at the 5′ end, respectively. A primer and probe set for an internal control gene, the bovine β-actin gene (β-actin set), was also used for bovine samples [24], and 5 pmol of each primer and 2.5 pmol of the probe were used per reaction. Both FAM-labeled and HEX-labeled probes for the bovine β-actin gene were prepared, and one of them was selected depending on the fluorophore of the other probe used in the multiplex assays. Probes of the Pan-PALV-1 and Pan-PALV-2 sets (PALV/S9/204P and PALV/S7/1067P) were labeled with minor groove binder-non-fluorescent quencher (MGB-NFQ) at the 3′ end, and the other probes were labeled with the dark quencher, BHQ-1, at the 3′ end. The conditions for the assays were as follows: 50 °C for 15 min for reverse transcription (RT), 95 °C for 2 min for inactivation of the RT enzyme and initial denaturation, 40 cycles of 95 °C for 15 s (denaturation), and 60 °C for 30 s (annealing and extension).

### 2.6. Evaluation of Analytical Specificity and Sensitivity of the Real-Time RT-PCR Assay

The analytical specificity of the assays was determined by using RNA samples extracted from 17 Japanese PALV strains, four Australian PALV strains, a Zimbabwean PALV strain and six other arboviruses (Akabane, Aino, Peaton, bluetongue, epizootic hemorrhagic disease and bovine ephemeral fever viruses). Viral RNA was extracted from the supernatant of the virus-infected cell cultures using a High Pure Viral RNA Kit (Roche Diagnostics, Basel, Switzerland). The RNA samples were tested in duplicate in multiplex assays with a combination of Pan-PALV-1 and Pan-PALV-2 sets and another combination of Chuzan- and D’Aguilar-specific sets, respectively.

In order to produce standard RNA for the real-time RT-PCR assay, artificial RNA templates were synthesized containing target sequences by molecular cloning and following in vitro transcription as previously described [25]. CHUV strain ON-1/E/02, DAGV strain ON-1/E/18 and Bunyip Creek virus (BCV) strain ON-14/E/17 were selected for the synthesis, and cDNA containing target sequences was amplified by RT-PCR with the QIAGEN OneStep RT-PCR Kit (Qiagen). The primers used for the RT-PCR are shown in Table 2. The products of the in vitro transcription were purified with an RNeasy Mini Kit (Qiagen), and the concentration and purity of the purified RNA were measured with a NanoDrop ND-1000 Spectrophotometer (NanoDrop Technologies, Wilmington, DE, USA). After the measurement, 10-fold dilutions were prepared as 1 × 10^9^ to 1 × 10^1^ copies/2 μL and used as standards for validation of the real-time RT-PCR assay.

The sensitivity of the assay was also examined by using spiked samples. To prepare spiked samples, 50 μL of propagated virus (cell culture supernatant; CHUV strain ON-1/E/02 at 1.58 × 10^6^ TCID_50_/mL, DAGV strain ON-1/E/18 at 1.32 × 10^5^ TCID_50_/mL, and BCV strain ON-14/E/17 at 1.00 × 10^6^ TCID_50_/mL) was added to 950 μL of EDTA-treated whole blood or brain homogenate of a healthy cow (PALV-free) and then mixed well. Next, the samples were centrifuged, and the supernatant was collected for RNA extraction. In parallel, 50 μL of each virus was mixed with 950 μL of Eagle’s MEM. Then, RNA was extracted from both the supernatant and the MEM using the High Pure Viral RNA Kit (Roche Diagnostics). It was then used as a template for the real-time RT-PCR assay.

### 2.7. Validation of the Real-Time RT-PCR Assay Using Field-Collected Samples

The diagnostic specificity of the assay was examined by using PALV-free bovine blood and tissue samples. RNA was extracted from 31 tissue samples from 22 cattle and 11 blood cell samples from 11 cattle using the High Pure Viral RNA kit (Roche Diagnostics) and used as templates for the real-time RT-PCR assay. The diagnostic sensitivity of the assay was examined using blood and tissue samples collected from stillborn fetuses and newborn calves with Chuzan disease-like congenital abnormalities in Japan from February 2019 to April 2019. A main feature in the stillborn fetuses was cerebral defects, and the main features in the calves were inability to stand, blindness and circling. Ten blood samples and 75 tissue samples from a total of 16 cattle were subjected to viral RNA extraction using High Pure Viral RNA Kit (Roche Diagnostics). The RNA samples were used as templates for the real-time RT-PCR assay, and all the samples were tested in duplicate.

## 3. Results

### 3.1. Genetic and Phylogenetic Characteristics of Seg-2

**Figure 1:**
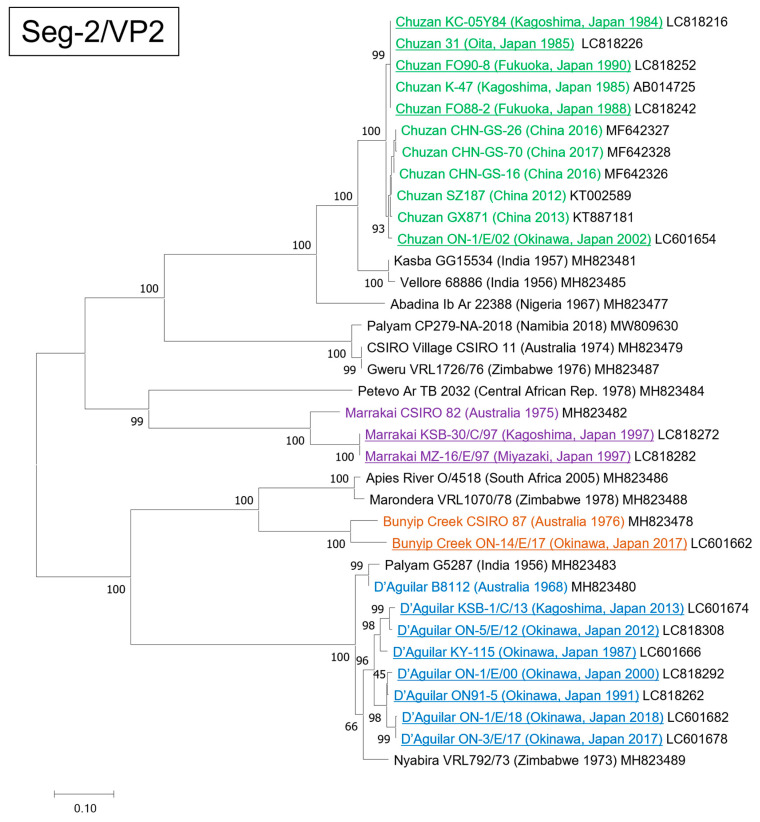
Phylogenetic profile showing the relationships among the Palyam serogroup virus (PALV) strains based on the complete coding region of genome segment 2. The Japanese PALV strains sequenced in this study are underlined. Serotypes Chuzan, Marrakai, Bunyip Creek, and D’Aguilar are shown in green, purple, brown, and blue, respectively. The percentage bootstrap values calculated from 1000 replications are indicated around the internal nodes. The scale represents 0.10% sequence divergence.

The nucleic acid and amino acid identities of Seg-2/VP2 among the Japanese PALV strains used in this study ranged from 52.24–99.96%, and 38.21–100%, respectively (Table S1), and the strains were classified into four groups in the phylogenetic tree constructed based on complete Seg-2 sequences (Figure 1). Five Japanese strains isolated between 1984 and 2002 (KC-05Y84, 31, FO88-2, FO90-8, and ON-1/E/02) were closely related to CHUV prototype strain K-47. The identities between strains ON-1/E/02 and K-47 were 98.07% and 98.70% at nucleotide and amino acid levels, respectively (Table 3). Seven other Japanese strains isolated from 1987 to 2018 (KY-115, ON91-5, ON-1/E/00, ON-5/E/12, KSB-1/C/13, ON-3/E/17, and ON-1/E/18) were closely related to Nyabira virus (NYAV) prototype strain VRL792/73 and DAGV prototype strain B8112. Identities at the nucleotide and amino acid levels were 91.77% and 96.37% between strains ON-1/E/18 and VRL792/73 and 91.60% and 94.45% between strains ON-1/E/18 and B8112, respectively (Table 3). Two other Japanese strains isolated in 1997 (KSB-30/C/97 and MZ-16/E/97) were closely related to the MARV prototype strain CSIRO 82, isolated in Australia in 1975, and identity between strains KSB-30/C/97 and CSIRO 82 were 87.34% and 89.92% at nucleotide and amino acid levels (Table 3). The other Japanese strain, ON-14/E/17, isolated in 2017, was closely related to the BCV prototype strain CSIRO 87, with identities of 89.97% and 94.23% at nucleotide and amino acid levels (Table 3).

### 3.2. Genetic and Phylogenetic Characteristics of the Other Genome Segments

Phylogenetic trees constructed based on Seg-1 and Seg-3 to Seg-10 are shown in Figures S1–S9. Most of the Japanese PALV strains sequenced in this study formed a group with Chinese and Indian PALV strains in the phylogenetic trees based on Seg-1, 3, 4, 5, 7, 8, and 9 (Figures S2–S5 and S7–S9). However, some of the Japanese PALV strains were most closely related to Australian PALV strains in Seg-7 and 10 (Figures S7 and S10). In Seg-7, the BCV strain ON-14/E/17 was closely related to BCV strain CSIRO 87, DAGV strain B8112, and CSIRO Village virus strain CSIRO 11, which were isolated in Australia between 1968 and 1976 (Figure S7). In Seg-10, the BCV strain ON-14/E/17 and MARV strains KSB-30/E/97 and MZ-16/E/97 were closely related to the three Australian strains mentioned above, the BCV strain CSIRO 87, the DAGV strain B8112, and the CSIRO Village virus strain CSIRO 11 (Figure S10). In Seg-6, the Japanese PALV strains were divided into two groups (Figure S6), and one of the groups consisting of Japanese CHUV and MARV strains was closely related to the Kasba strain GG15534 and the Vellore strain 68886. The other group, consisting of Japanese DAGV and BCV strains, was closely related to the NYAV strain VRL792/73 and the DAGV strain B8112.

### 3.3. Analytical Specificity and Sensitivity of the Real-Time RT-PCR Assay

All the Japanese PALV strains used in the present study tested positive in the real-time RT-PCR assay with the Pan-PALV-1 and Pan-PALV-2 sets. The *Ct* values using the Pan-PALV-1 and Pan-PALV-2 sets ranged 15.23–19.98 and 17.93–23.02 for CHUV, 18.94–21.02 and 18.05–22.47 for DAGV, 18.98–19.76 and 27.05–30.08 for BCV, and 15.35–20.12 and 14.83–19.91 for MARV, respectively. All the Australian PALV strains tested positive with the Pan-PALV-1 set, and the *Ct* values were 15.75–19.48, but the Zimbabwean PALV strain, NYAV strain 792/73, tested negative. On the other hand, all the Australian and the Zimbabwean PALV strains tested positive with the Pan-PALV-2 set except the CSIRO Village virus strain CSIRO 11, and the *Ct* values were 16.37–30.31. All the CHUV and DAGV strains used in the present study tested positive with the Chuzan- and D’Aguilar-specific sets, respectively. The *Ct* values using the Chuzan-specific set ranged from 15.17–19.16 for the CHUV strains, and an Australian virus strain DPP66, whose serotype has not been identified but is closely related to serotype Kasba, had a *Ct* value of 22.83. The *Ct* values using the D’Aguilar-specific set ranged from 20.21 to 21.78 for Japanese DAGV strains, and the prototype strain B8112 had a *Ct* value of 35.88. The NYAV strain 792/73 also tested positive with the D’Aguilar-specific set and had a *Ct* value of 22.24. All the following arboviruses other than PALV tested negative in the real-time RT-PCR assay: Akabane virus, Aino virus, Peaton virus, bluetongue virus (BTV), epizootic hemorrhagic disease virus (EHDV), bovine ephemeral fever virus (Table 4). The concentration of the RNA templates was 72.3–136.0 ng/μL.

**Figure 2:**
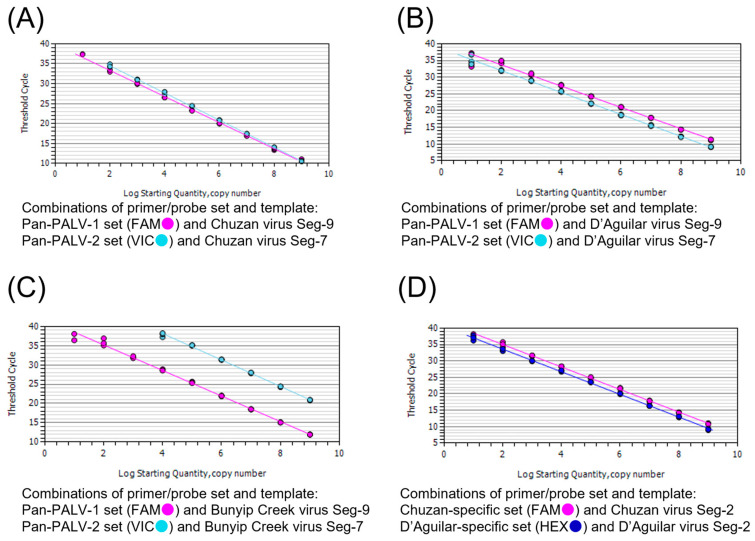
Standard curves for artificial RNA templates of Chuzan, D’Aguilar, and Bunyip Creek viruses (CHUV, DAGV, and BCV). Ten-fold serial dilutions (10^9^–10^1^ copies/tube) of the templates were subjected to the real-time RT-PCR assay. The amplification slopes, correlation coefficient, and efficiency of amplification are as follows: (**A**) −3.271, 0.998, and 102.2% (Pan-PALV-1 set for CHUV Seg-9 gene), −3.410, 1.000, 96.4% (Pan-PALV-2 set for CHUV Seg-7 gene). (**B**) −3.187, 0.991, and 106.0% (Pan-PALV-1 set for DAGV Seg-9 gene), −3.286, 0.997, and 101.5% (Pan-PALV-2 set for DAGV Seg-7 gene). (**C**) −3.318, 0.995, and 100.1% (Pan-PALV-1 set for BCV Seg-9 gene), −3.441, 0.998, and 95.3% (Pan-PALV-2 set for BCV Seg-7 gene). (**D**) −3.429, 0.999, and 95.7% (Chuzan-specific set for CHUV Seg-2 gene), −3.447, 0.998, and 95.0% (D’Aguilar-specific set for DAGV Seg-2 gene).

The detection limits of the assay using the Pan-PALV-1 and Pan-PALV-2 sets were 100 copies/tube for CHUV and BCV and 10 copies/tube for DAGV, respectively (Figure S11). The detection limits of the assay using Chuzan- and D’Aguilar-specific sets were 100 and 10 copies/tube for CHUV and DAGV, respectively (Figure S11). Standard curves were generated (Figure 2), and the amplification slopes, correlation coefficient, and efficiency of amplification were calculated as follows; −3.271, 0.998 and 102.2% (Pan-PALV-1 set for CHUV Seg-9 gene), −3.410, 1.000, 96.4% (Pan-PALV-2 set for CHUV Seg-7 gene), −3.187, 0.991 and 106.0% (Pan-PALV-1 set for DAGV Seg-9 gene), −3.286, 0.997 and 101.5% (Pan-PALV-2 set for DAGV Seg-7 gene), −3.318, 0.995 and 100.1% (Pan-PALV-1 set for BCV Seg-9 gene), −3.441, 0.998 and 95.3% (Pan-PALV-2 set for BCV Seg-7 gene), −3.429, 0.999 and 95.7% (Chuzan-specific set for CHUV Seg-2 gene), −3.447, 0.998 and 95.0% (D’Aguilar-specific set for DAGV Seg-2 gene).

The samples spiked with CHUV strain ON-1/E/02 tested positive using Pan-PALV-1, Pan-PALV-2 and Chuzan-specific sets, and the sample spiked with DAGV strain ON-1/E/18 tested positive using Pan-PALV-1, Pan-PALV-2 and D’Aguilar-specific sets. The samples spiked with BCV strain ON-14/E/17 tested positive using the Pan-PALV-1, while the strain tested negative using the Pan-PALV-2 set. In each of the spiked samples that tested positive, the *Ct* value was close to that obtained by testing MEM mixed with the same titer of the virus, and the difference in *Ct* values between the two samples was within the range of 0.02–1.38 (Table 5). The concentration of the RNA was 155.3–247.7 ng/μL.

### 3.4. Diagnostic Specificity and Sensitivity of the Real-Time RT-PCR Assay

All the PALV-free tissue homogenate and blood cells tested negative using the Pan-PALV-1, Pan-PALV-2, Chuzan-specific, and D’Aguilar-specific sets. On the other hand, all the samples tested positive using the β-actin set, and the *Ct* values were 19.96–31.64. The concentration of the RNA was 61.3–969.2 ng/μL.

The real-time RT-PCR assay was performed using the field samples collected from stillborn fetuses and newborn calves with congenital abnormalities, and 22, 34, and 29 samples tested positive using Pan-PALV-1, Pan-PALV-2, and D’Aguilar-specific sets, respectively. The tissue homogenate and blood cells that tested positive using the Pan-PALV-1 set had *Ct* values of 29.75–37.48 and 29.81–37.48, and *Ct* values obtained using the Pan-PALV-2 set were 29.99–38.51 and 30.33–37.37, respectively. For tissue homogenate and blood cells that tested positive using the D’Aguilar-specific set, *Ct* values were 28.42–38.26 and 28.94–38.43, respectively. All the samples collected from the stillborn fetuses and newborn calves tested negative using a Chuzan-specific set. Of the 16 cattle tested, one tested positive for PALV only, another tested positive for DAGV only, and the remaining 14 tested positive for both PALV and DAGV (Table 6). The concentration of the RNA was 50.0–830.5 ng/μL.

## 4. Discussion

Genetic analysis of the complete coding region of Seg-2 in the present study revealed that the Japanese PALV strains were classified into four serotypes: CHUV, DAGV, and BCV, which we have previously identified [7,26], plus MARV. MARV is a member of PALV, identified in Australia in 1974–1976 from mosquitoes and *Culicoides* biting midges [27]. Our data suggest that MARV had been introduced to Asia in the past and was present in Japan at least in 1997. Although it has been suggested that MARV isolated from *Culicoides* biting midges in Australia in 1975 may have resulted from the reassortment of some PALVs in Australia and India [11] to our knowledge, this is the first case of MARV isolation outside Australia and the first case of MARV isolation from cattle.

Genetic analysis of the complete coding region of Seg-2 in the present study also revealed that seven Japanese PALV strains isolated in 1987–2018 showed slightly higher identities to the NYAV prototype strain VRL792/73 (95.66–96.67%) than to the DAGV prototype strains B8112 (94.45–94.85%) at the amino acid level. Considering these identities, the seven Japanese PALV strains may be classified as NYAV; however, to begin with, the prototype strains of NYAV and DAGV are highly identical at VP2, with 95.96% identity. If multiple virus strains show such a high identity, they can be classified as the same serotype in another orbivirus, epizootic hemorrhagic disease virus [28]. In addition, the VP2 identity between CHUV and Kasba virus, which were reported to be serologically identical [3], is 95.50%. Therefore, the seven Japanese PALV strains analyzed in this study, KY-115, ON91-5, ON-1/E/00, ON-5/E/12, KSB-1/C/13, ON-3/E/17, and ON-1/E/18, could be classified as either NYAV or DAGV. However, the seven Japanese PALV strains were more closely related to other Asian/Australian PALV strains, not African PALV strains, in the phylogenetic analysis based on all the genome segments other than Seg-2 and 6. In addition, the prototype strains of NYAV and DAGV were sorted into Afrotropical and Australasian groups in the phylogenetic analysis based on the concatenated dataset, respectively [11]; thus, it would be more appropriate to classify the seven Japanese PALV strains as DAGV rather than NYAV.

Our data obtained by WGS suggest that PALV forms a gene pool in Japan, China, and India and has occasionally caused reassortment between Asian and Australian PALVs in the past. The Japanese PALV strains sequenced in this study and Chinese PALV strains were closely related to the Kasba and Vellore viruses isolated in India in 1956–1957 in genome segments encoding viral proteins other than outer-capsid. On the other hand, for Seg-7 and/or 10, some Japanese strains, BCV strain ON-14/E/17 and MARV strains KSB-30/C/97 and MZ-16/E/97, are closely related to Australian PALV strains isolated in 1968–1976: the CSIRO Village virus strain CSIRO 11, the BCV strain CSIRO 87, and the DAGV strain B8112. Therefore, it is presumed that these Japanese BCV and MARV strains are reassortant viruses that have acquired Seg-7 and/or Seg-10 as well as Seg-2 from the Australian PALV strains. Another characteristic of the Japanese PALV strains revealed by the WGS was that they formed two groups with partial correlations of serotypes in the phylogenetic tree based on Seg-6 (Figure S6). This result was similar to that of the phylogenetic analysis of Seg-6 in Japanese BTV and EHDV strains [17,29].

The newly developed real-time RT-PCR assay is capable of broad detection of PALV and specific detection of CHUV and DAGV, and it has sufficient sensitivity for diagnosis. Using this assay, the DAGV gene was detected in blood and tissue samples collected from bovine stillborn fetuses and calves with congenital abnormalities in Japan from February 2019 to April 2019. In the area where the samples were collected, seroconversion to DAGV was observed in sentinel cattle in the summer of 2018 [30], suggesting that DAGV infection of dams may have caused stillbirths and congenital abnormalities in calves. Since congenital abnormalities in calves occur approximately five months after CHUV infection in the dams in Chuzan disease [4,5,6], it is assumed that DAGV also has a similar time lag between infection and disease occurrence. It is thus difficult to isolate CHUV or DAGV or to detect viral genes by conventional RT-PCR assays from the affected fetuses and calves. All the bovine fetuses and calves sampled in this study tested negative for PALV by a conventional multiplex RT-PCR assay for arboviruses [7]; however, they tested positive for PALV and/or DAGV by the newly developed real-time RT-PCR assay. To our knowledge, this is the first report of PALV gene detection in bovine stillborn fetuses or calves with congenital abnormalities.

**Figure 3:**
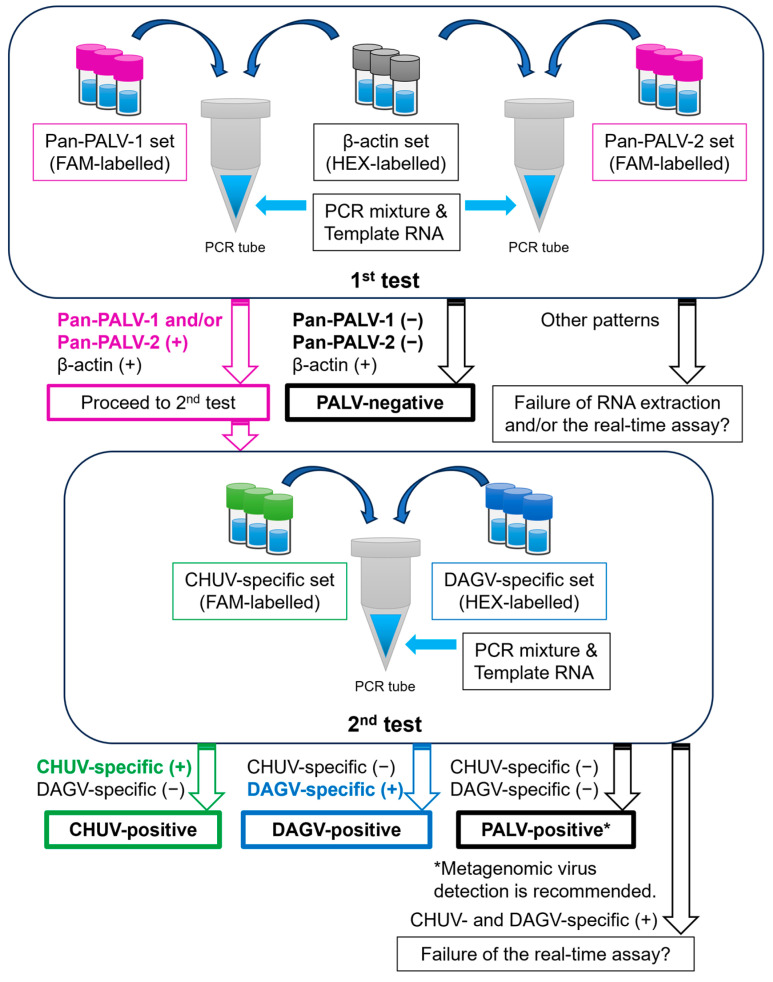
Proposed use of the real-time RT-PCR assay for the detection of targeted viruses. Samples are tested with combinations of Pan-PALV-1 and β-actin sets and Pan-PALV-2 and β-actin sets in multiplex assays (first test). When samples tested positive for Palyam serogroup virus, the samples were then subjected to the second test with a combination of Chuzan- and D’Aguilar-specific sets in a multiplex assay.

The proposed use of the real-time RT-PCR assay is shown in Figure 3. First, a multiplex real-time RT-PCR assay is performed using Pan-PALV-1 and β-actin sets and Pan-PALV-2 and β-actin sets, respectively (first test). If samples test positive for PALV, a multiplex real-time RT-PCR assay is performed using Chuzan-specific and D‘Aguilar-specific sets as a second test to check the presence of CHUV and DAGV. If a sample tests positive for PALV by the 1st test but negative for CHUV or DAGV by the second test, metagenomic virus detection is recommended. Since much remains unknown about the risk of clinical disease due to PALV infection in cattle, it is expected that this assay will help to further clarify the actual status of clinical cases caused by PALV.

## 5. Conclusions

This study provides a comprehensive genetic characterization of PALVs isolated in Japan from 1984 to 2018, revealing the presence of MARV outside Australia for the first time and the possibility of reassortment events among PALVs in the Asia-Pacific region. In addition, we developed a sensitive and specific real-time RT-PCR assay capable of broad detection of PALV and specific identification of CHUV and DAGV. This assay successfully detected DAGV in bovine stillborn fetuses and congenitally abnormal calves, marking a significant improvement over a conventional RT-PCR assay. These findings enhance our understanding of PALV epidemiology and evolution, providing crucial tools for better diagnosis of PALV-related diseases in ruminants.

## Figures and Tables

**Table 1:** Characteristics of Japanese PALV strains used for whole-genome sequencing in this study.

Strain	Year Collected	Geographical Origin (Prefecture)	Source	Serotype
KC-05Y84	1984	Kagoshima	*Culicoides oxystoma*	Chuzan
31	1985	Oita	bovine erythrocytes	Chuzan
KY-115	1987	Okinawa	bovine erythrocytes	D’Aguilar
FO-88-2	1988	Fukuoka	*Culex* spp.	Chuzan
FO-90-8	1990	Fukuoka	*Culex* spp.	Chuzan
ON91-5	1991	Okinawa	bovine erythrocytes	D’Aguilar
KSB-30/C/97	1997	Kagoshima	*Culicoides* spp.	Marrakai ^a^
MZ-16/E/97	1997	Miyazaki	bovine erythrocytes	Marrakai ^a^
ON-1/E/00	2000	Okinawa	bovine erythrocytes	D’Aguilar
ON-1/E/02	2002	Okinawa	bovine erythrocytes	Chuzan
ON-5/E/12	2012	Okinawa	bovine erythrocytes	D’Aguilar
KSB-1/C/13	2013	Kagoshima	*Culicoides* spp.	D’Aguilar
ON-3/E/17	2017	Okinawa	bovine erythrocytes	D’Aguilar
ON-14/E/17	2017	Okinawa	bovine erythrocytes	Bunyip Creek
ON-1/E/18	2018	Okinawa	bovine erythrocytes	D’Aguilar

^a^ Identified by phylogenetic analysis based on Seg-2 and calculation of nucleotide and amino acid identities of Seg-2/VP2 among PALV strains.

**Table 2:** Primers and probes used for the development of the real-time RT-PCR assay.

Set	Oligo Name	Sequence (5′-3′)	Target	Genome Position
Primers and probes for the real-time RT-PCR assay				
Pan-PALV-1	PALV/S9/182F	GGTGGAGGCGGAGAGAATAAA	Palyam serogroup	182–202 (Seg-9)
	PALV/S9/246R	TTAGCCTCTGTTGTGCGATCTG		225–246
	PALV/S9/204P ^a^	AAAAGGGAGGAGATGAAAGT		204–223
Pan-PALV-2	PALV/S7/1047F	GGCGCAAGCGTACAGATGA	Palyam serogroup	1047–1065 (Seg-7)
	PALV/S7/1109R	TCTAGTGTGACTGATGCATTGTGAA		1085–1109
	PALV/S7/1067P ^a^	CGGTGTTGCATGGCA		1067–1081
Chuzan- specific	CHUV/S2/316F	CTCGTAAAGGACCAAGAAATAAACCT	Chuzan	316–341 (Seg-2)
	CHUV/S2/443R	ACCGCACTTGTAGTCTCTAAACGAT		419–443
	CHUV/S2/365P	TGAACATATACCAACGTCAATACAGGCGGA		365–394
D’Aguilar- specific	DAGV/S2/10F	TCGCAGGATGGACGAGTTTT	D’Aguilar	10–29 (Seg-2)
	DAGV/S2/99R	CTAACAACGATCTCATGTTGCAAA		76–99
	DAGV/S2/49P	CTTCGATACTCAGCGATTGGCCCAAA		49–74
β-actin	ACT-1005-F	CAGCACAATGAAGATCAAGATCATC	β-actin (bovine)	
	ACT-1135-R	CGGACTCATCGTACTCCTGCTT		
	ACT-1081-Probe	TCGCTGTCCACCTTCCAGCAGATGT		
Primers for molecular cloning				
PALV/S9	PALV/S9/7F	AAAGTTGTGGTTGATGACG	Palyam serogroup	7–25 (Seg-9)
	PALV/S9/352R	TCCAATCTCTGTTCCTGTTC		333–352
PALV/S7	PALV/S7/1021F	GTTGCACCACAGAATAGAG	Palyam serogroup	1021–1039 (Seg-7)
	PALV/S7/1131R	CGTGCTAACACTAAATACC		1113–1131
CHUV/S2	CHUV/S2/256F	GGGATGAAGGAAGGAGAAC	Chuzan	256–274 (Seg-2)
	CHUV/S2/470R	GCCTACATAAGGTTCAACGC		451–470
DAGV/S2	DAGV/S2/1F	GTTAAATTTTCGCAGGATGG	D’Aguilar	1–20 (Seg-2)
	DAGV/S2/185R	GCTCCTCATTCCATACAACC		166–185

^a^ Minor groove binder (MGB) probes.

**Table 3:** Percentage sequence identities of Seg-2/VP2 between prototype strains and four Japanese PALV strains (Chuzan ON-1/E/02, Marrakai KSB-30/C/97, Bunyip Creek ON-14/E/17, and D’Aguilar ON-1/E/18) at nucleotide (NT) and amino acid (AA) levels.

	[Chuzan] ON-1/E/02 (Okinawa, Japan 2002)		[Marrakai] KSB-30/C/97 (Kagoshima, Japan 1997)		[Bunyip Creek] ON-14/E/17 (Okinawa, Japan 2017)		[D’Aguilar] ON-1/E/18 (Okinawa, Japan 2018)	
	NT	AA	NT	AA	NT	AA	NT	AA
[Chuzan] K-47 (Kagoshima, Japan 1985)	98.07	98.70	55.29	44.53	53.96	41.00	52.49	40.35
[Kasba] GG15534 (India 1957)	90.16	95.50	55.61	43.94	53.83	41.20	52.26	40.55
[Vellore] 6888 (India 1956)	89.10	93.61	55.05	43.25	53.33	40.71	52.31	40.01
[Abadina] Ib Ar 22388 (Nigeria 1967)	80.29	86.52	55.82	43.34	53.50	39.52	52.62	39.00
[CSIRO Village] CSIRO 11 (Australia 1974)	59.44	53.00	56.08	44.87	53.41	39.13	53.06	37.19
[Gweru] VRL1726/76 (Zimbabwe 1976)	59.44	53.00	56.11	44.87	53.57	39.13	53.00	37.19
[Marrakai] CSIRO 82 (Australia 1975)	55.29	44.22	87.34	89.92	54.43	39.92	53.13	38.38
[Petevo] Ar TB 2032 (Central African Rep. 1978)	54.34	44.95	58.62	51.72	52.90	39.60	52.37	37.08
[Bunyip Creek] CSIRO 87 (Australia 1976)	54.46	42.19	53.20	39.98	89.97	94.23	56.76	47.32
[Marondera] VRL1070/78 (Zimbabwe 1978)	52.89	40.92	53.41	39.51	71.47	74.01	56.21	46.38
[Apies River] O/4518 (South Africa 2005)	52.60	41.11	53.37	39.31	71.24	73.91	56.69	46.68
[D’Aguilar] B8112 (Australia 1968)	52.73	40.35	52.95	38.34	56.58	47.42	91.60	94.45
[Nyabira] VRL792/73 (Zimbabwe 1973)	52.37	40.25	53.05	38.44	56.18	47.71	91.77	96.37
[Palyam] G5287 (India 1956)	52.57	39.46	52.65	37.62	56.22	46.23	89.64	91.23

**Table 4:** *Ct* values of the real-time PCR assay obtained by testing PALV strains and other arboviruses using the four primer/probe sets.

Serotype/Virus	Strain	Year Collected	Geographical Origin	*Ct* Value			
				Pan-PALV Set-1	Pan-PALV Set-2	CHUV- Specific Set	DAGV- Specific Set
Chuzan (Kasba)	KC-05Y84	1984	Kagoshima	17.78	20.72	16.93	– ^a^
	31	1985	Oita	17.67	20.81	16.90	–
	FO88-2	1988	Fukuoka	18.93	21.90	18.12	–
	FO90-8	1990	Fukuoka	19.98	23.02	19.16	–
	ON-1/E/02	2002	Okinawa	15.23	17.93	15.17	–
(Not typed)	DPP66 ^b^	1981	Australia	15.75	26.92	22.83	–
D’Aguilar	KY-115	1987	Okinawa	19.97	18.05	–	20.73
	ON91-5	1991	Okinawa	19.42	18.81	–	21.22
	ON-1/E/00	2000	Okinawa	18.94	21.86	–	21.17
	KSB-29/E/01 ^c^	2001	Kagoshima	19.34	22.47	–	21.35
	ON-5/E/12	2012	Okinawa	19.12	19.08	–	21.61
	KSB-1/C/13	2013	Kagoshima	19.64	19.29	–	21.78
	ON-3/E/17	2017	Okinawa	21.02	19.79	–	21.71
	ON-1/E/18	2018	Okinawa	20.23	18.75	–	20.21
	B8112	1968	Australia	19.48	18.28	–	35.88
Nyabira	792/73	1973	Zimbabwe	–	21.74	–	22.24
Bunyip Creek	CSIRO 58	1976	Australia	17.45	16.47	–	–
	ON-1/E/08 ^d^	2008	Okinawa	19.76	27.05	–	–
	ON-14/E/17	2017	Okinawa	18.98	30.08	–	–
CSIRO Village	CSIRO 11	1974	Australia	16.90	–	–	–
Marrakai	CSIRO 82	1975	Australia	19.36	30.31	–	–
	KSB-30/C/97	1997	Kagoshima	15.35	14.83	–	–
	MZ-16/E/97	1997	Miyazaki	20.12	19.91	–	–
Akabane	OBE-1	1974	Okayama	–	–	–	–
Aino	JaNAr28	1964	Nagasaki	–	–	–	–
Peaton	KSB-1/P/06	2006	Kagoshima	–	–	–	–
Bluetongue	TO2-1	1994	Tochigi	–	–	–	–
Epizootic hemorrhagic disease	Ibaraki No. 2	1959	Ibaraki	–	–	–	–
Bovine ephemeral fever	YHL	1966	Yamaguchi	–	–	–	–

^a^ No *Ct*. ^b^ Isolated from bovine blood. Most closely related to serotype Kasba. GenBank accession No. AB034667 (Seg-7). ^c^ Isolated from bovine erythrocyte. GenBank accession No. AB177630 (Seg-2). ^d^ Isolated from bovine erythrocyte. GenBank accession No. AB973440 (Seg-2).

**Table 5:** *Ct* values of the real-time PCR assay obtained by testing spiked samples and MEM mixed with Chuzan virus, D’Aguilar virus or Bunyip Creek virus.

Sample	*Ct* Value			
	Pan-PALV Set-1	Pan-PALV Set-2	CHUV- Specific Set	DAGV- Specific Set
Chuzan virus strain ON-1/E/02				
Spiked (brain homogenate)	17.18	22.68	18.93	– ^a^
Spiked (blood cells)	17.30	22.47	18.75	–
MEM	17.28	23.80	18.46	–
D’Aguilar virus strain ON-1/E/18				
Spiked (brain homogenate)	20.38	20.35	–	21.40
Spiked (blood cells)	21.20	20.73	–	20.71
MEM	20.26	20.32	–	20.03
Bunyip Creek virus strain ON-14/E/17				
Spiked (brain homogenate)	21.46	–	–	–
Spiked (blood cells)	22.68	–	–	–
MEM	21.73	–	–	–

^a^ No *Ct*.

**Table 6:** *Ct* values of the real-time PCR assay obtained by testing field-collected bovine samples.

Cattle No.	Sample	*Ct* Value ^a^		
		Pan-PALV Set-1	Pan-PALV Set-2	DAGV- Specific Set
1	Cerebellum	– ^b^	37.03	–
	Thoracic spinal cord	–	36.78	–
	Lumbar spinal cord	–	36.07	–
	Blood cells	34.48	33.65	33.94
	Cervical spinal cord	–	–	–
2	Cerebellum	36.25	36.81	36.55
	Cervical spinal cord	–	37.60	–
	Blood cells	33.90	33.99	33.62
	Others (thoracic spinal cord, lumbar spinal cord)	–	–	–
3	Cerebellum and brain stem	36.09	35.18	34.89
	Blood cells	30.74	31.04	29.97
	Others (cervical spinal cord, thoracic spinal cord, lumbar spinal cord)	–	–	–
4	Cerebellum and brain stem	35.81	35.99	–
	Blood cells	36.69	35.94	–
	Others (cervical spinal cord, thoracic spinal cord, lumbar spinal cord)	–	–	–
5	Cerebellum and brain stem	32.21	33.07	33.83
	Others (cervical spinal cord, thoracic spinal cord)	–	–	–
6	Blood cells	32.34	32.65	31.94
	Lumbar spinal cord	–	–	–
7	Cerebellum and brain stem	–	–	38.11
	Cervical spinal cord	–	36.62	–
	Blood cells	–	37.37	35.96
	Others (thoracic spinal cord, lumbar spinal cord)	–	–	–
8	Cervical spinal cord	–	37.56	–
	Blood cells	36.32	36.49	36.05
	Others (thoracic spinal cord, lumbar spinal cord)	–	–	–
9	Blood cells	–	34.66	32.01
	Others (cerebellum, brain stem, cervical spinal cord, thoracic spinal cord, lumbar spinal cord)	–	–	–
10	Cerebrum	32.65	32.58	31.73
	Others (cerebellum, brain stem, spinal cord)	–	–	–
11	Cerebellum	36.38	35.92	34.57
	Blood cells	29.81	30.33	28.94
	Others (cerebrum, brain stem, cervical spinal cord, thoracic spinal cord, lumbar spinal cord)	–	–	–
12	Blood cells	–	–	38.43
	Others (cerebrum, cerebellum, brain stem, cervical spinal cord, thoracic spinal cord, lumbar spinal cord)	–	–	–
13	Cerebrum	–	36.73	36.18
	Medulla oblongata	38.23	37.20	–
	Heart	–	–	37.50
	Lung	29.75	29.99	28.42
	Liver	36.39	–	35.93
	Kidney	37.48	37.75	36.41
	Spleen	32.49	33.00	31.33
	Spinal cord	–	–	–
14	Heart	–	38.38	–
	Lung	33.99	34.04	32.46
	Liver	37.26	36.71	36.70
	Kidney	–	38.06	38.17
	Spleen	32.38	33.03	30.80
	Others (cerebrum, medulla oblongata)	–	–	–
15	Cerebrum	34.93	36.98	35.66
	Lung	–	–	–
16	Cerebrospinal fluid	–	37.73	37.13
	Spleen	–	–	38.26
	Placenta	–	38.51	35.73
	Others (cerebrum, cerebellum, medulla oblongata, spinal cord, heart, lung, liver, spleen, kidney)	–	–	–

^a^ All the samples tested negative using the CHUV-specific set. ^b^ No *Ct.*

## Data Availability

The data will be made available by the authors on request.

## Supplementary Materials

The following supporting information can be downloaded at: https://www.mdpi.com/article/10.3390/pathogens13070550/s1, Figure S1: Locations where the Japanese PALV strains used in this study were obtained. Figures S2–S10: Phylogenetic profile based on the coding region of genome segments 1, 3, 4, 5, 6, 7, 8, 9, and 10 showing the relationships between the PALV strains sequenced in this study and PALV strains isolated elsewhere; Figure S11: Amplification chart of the real-time RT-PCR assay obtained by testing artificial RNA templates of CHUV, DAGV, and BCV. Table S1: Percentage sequence identities of Seg-2/VP2 among Japanese strains of PALV at nucleotide and amino acid levels.
