# Supplementary material for: Genetic Characterization of Palyam Serogroup Viruses Isolated in Japan from 1984 to 2018 and Development of a Real-Time RT-PCR Assay for Broad Detection of Palyam Serogroup Viruses and Specific Detection of Chuzan (Kasba) and D’Aguilar Viruses
Source: Pathogens. 2024 Jun 28;13(7):550. doi: 10.3390/pathogens13070550 (PMC11279806; doi:10.3390/pathogens13070550)
Supplement: Supplementary file 1 [file pathogens-13-00550-s001.zip › Figs. S1-S11_Shirafuji et al.R1_18Jun.2024.pdf]

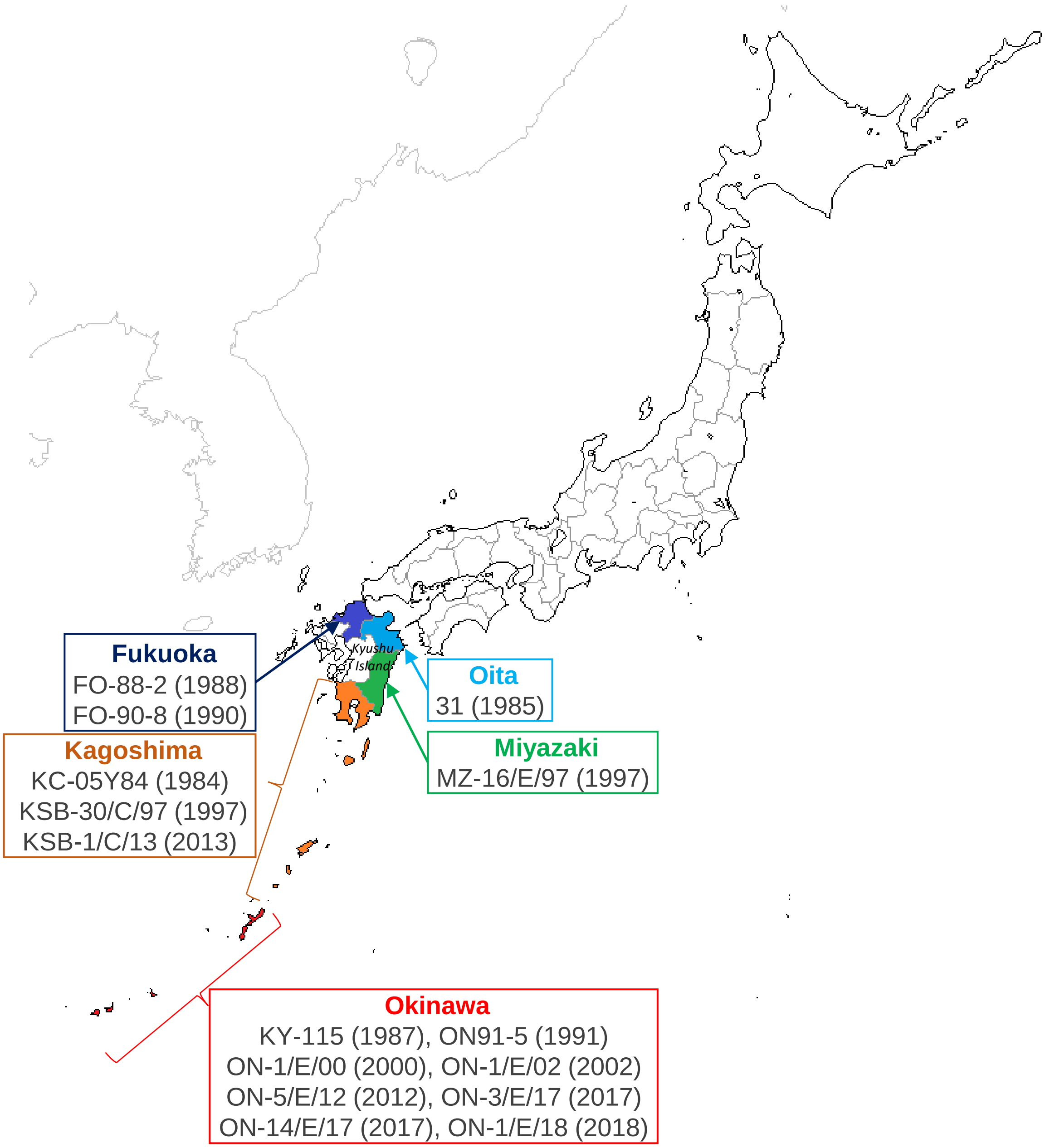

Figure S1. Locations where the Japanese PALV strains used in this study were obtained. This figure was created using a white map on a website that provides white map materials, Craft MAP (http://www.craftmap.box-i.net/).

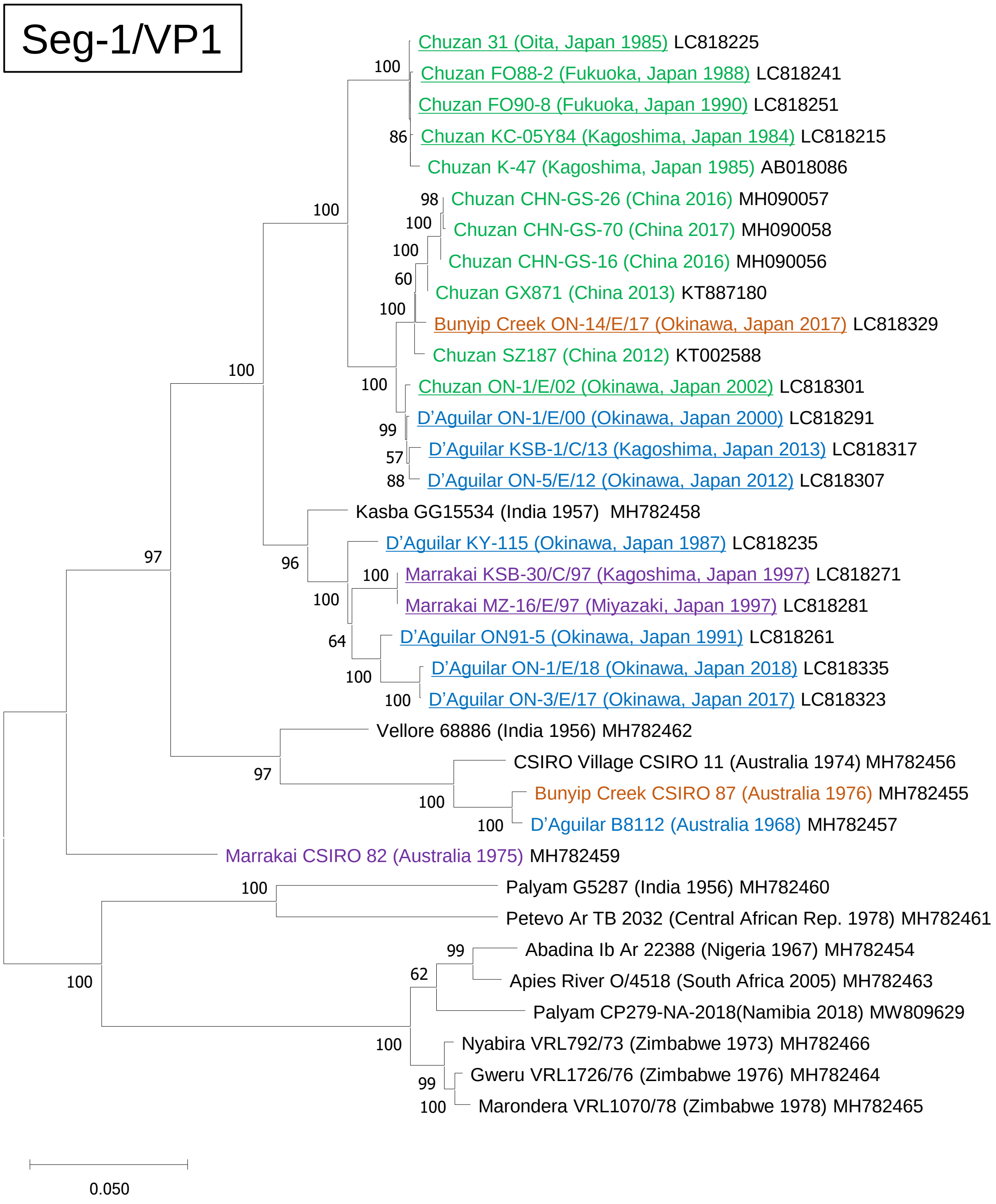

Figure S2. Phylogenetic profile showing the relationships among the Palyam serogroup virus (PALV) strains based on the complete coding region of genome segment 1. The Japanese PALV strains sequenced in this study are underlined. The percentage bootstrap values calculated from 1,000 replications are indicated around the internal nodes. The scale represents 0.05% sequence divergence.

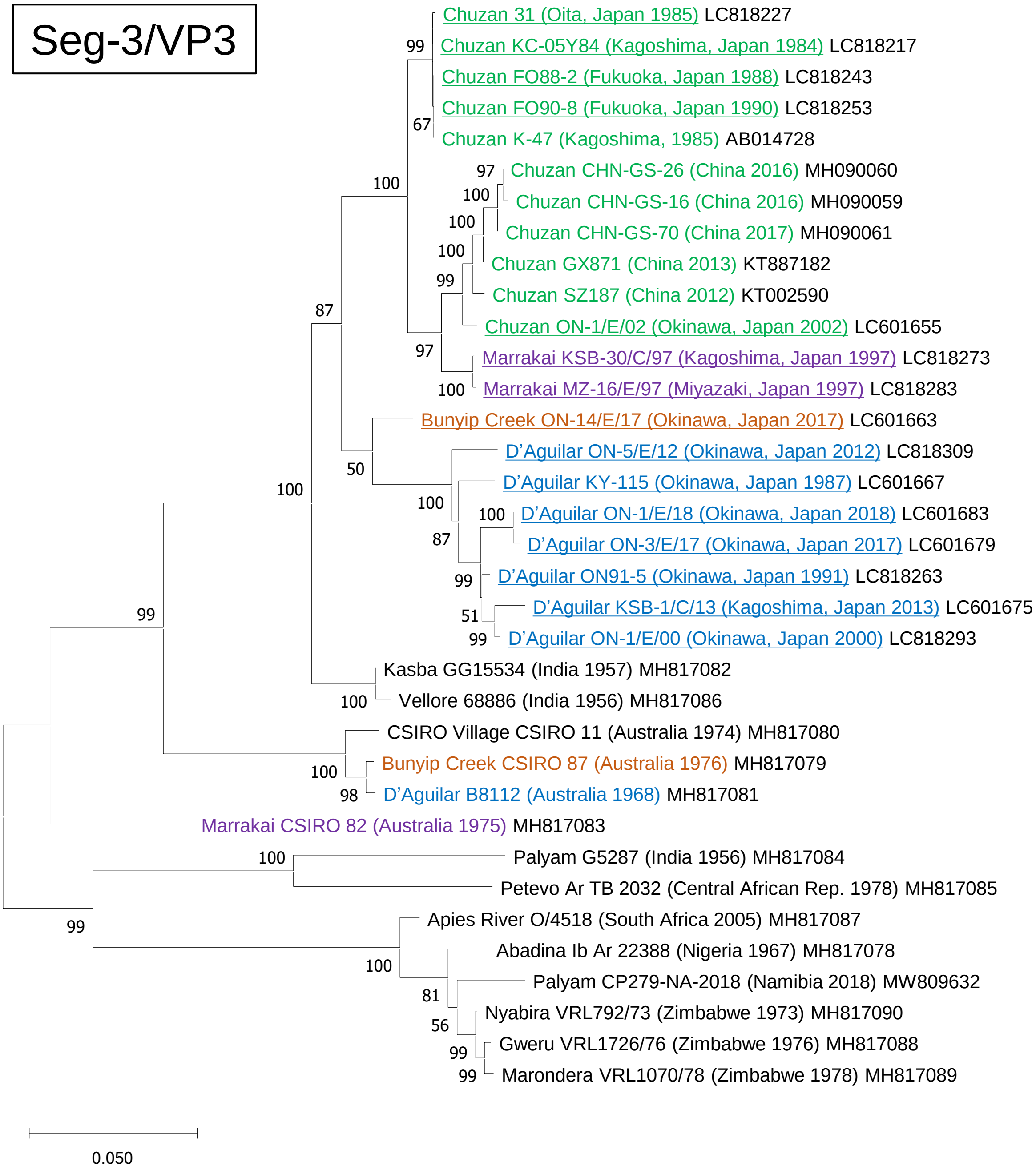

Figure S3. Phylogenetic profile showing the relationships among the Palyam serogroup virus (PALV) strains based on the complete coding region of genome segment 3. The Japanese PALV strains sequenced in this study are underlined. The percentage bootstrap values calculated from 1,000 replications are indicated around the internal nodes. The scale represents 0.05% sequence divergence.

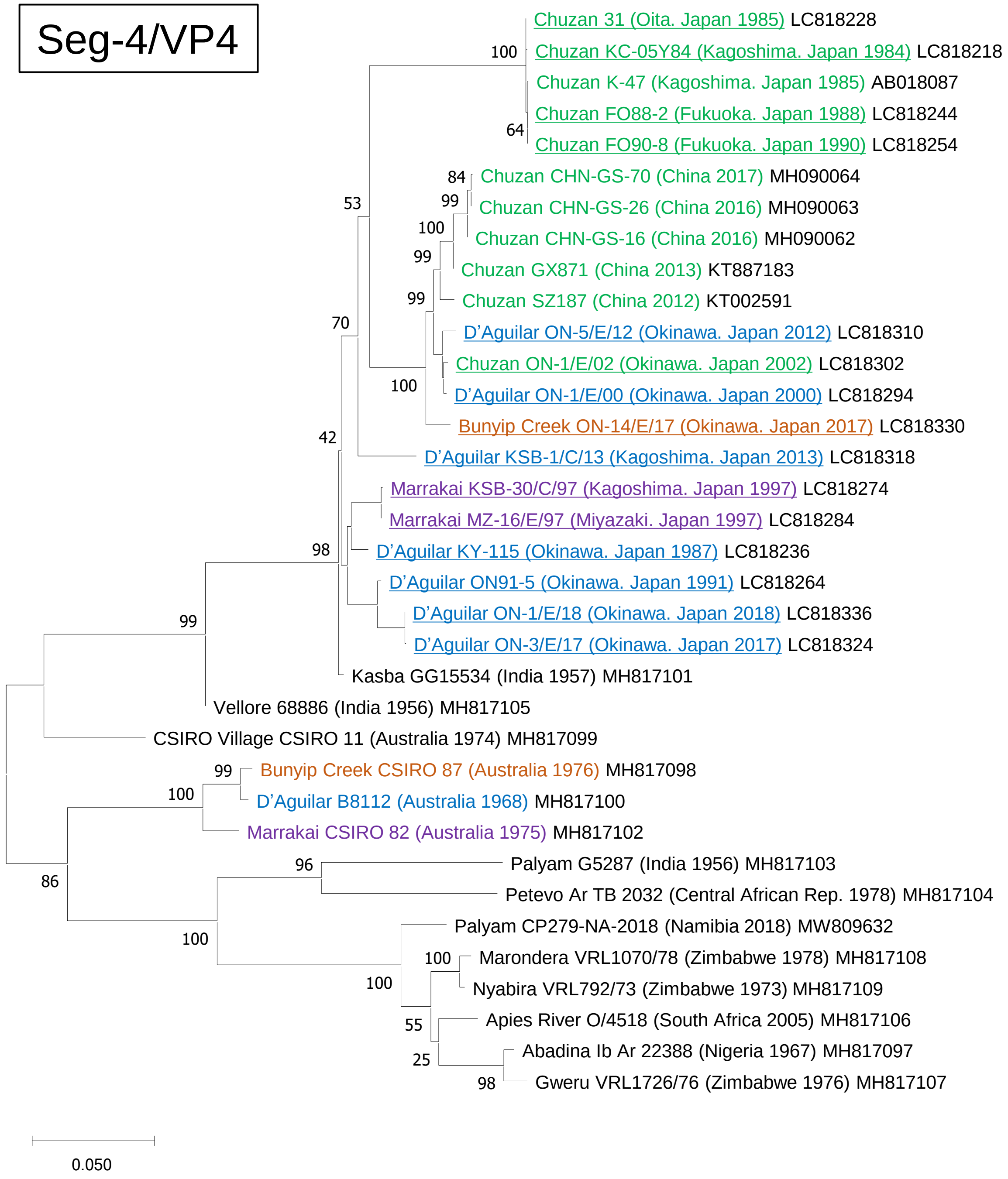

Figure S4. Phylogenetic profile showing the relationships among the Palyam serogroup virus (PALV) strains based on the complete coding region of genome segment 4. The Japanese PALV strains sequenced in this study are underlined. The percentage bootstrap values calculated from 1,000 replications are indicated around the internal nodes. The scale represents 0.05% sequence divergence.

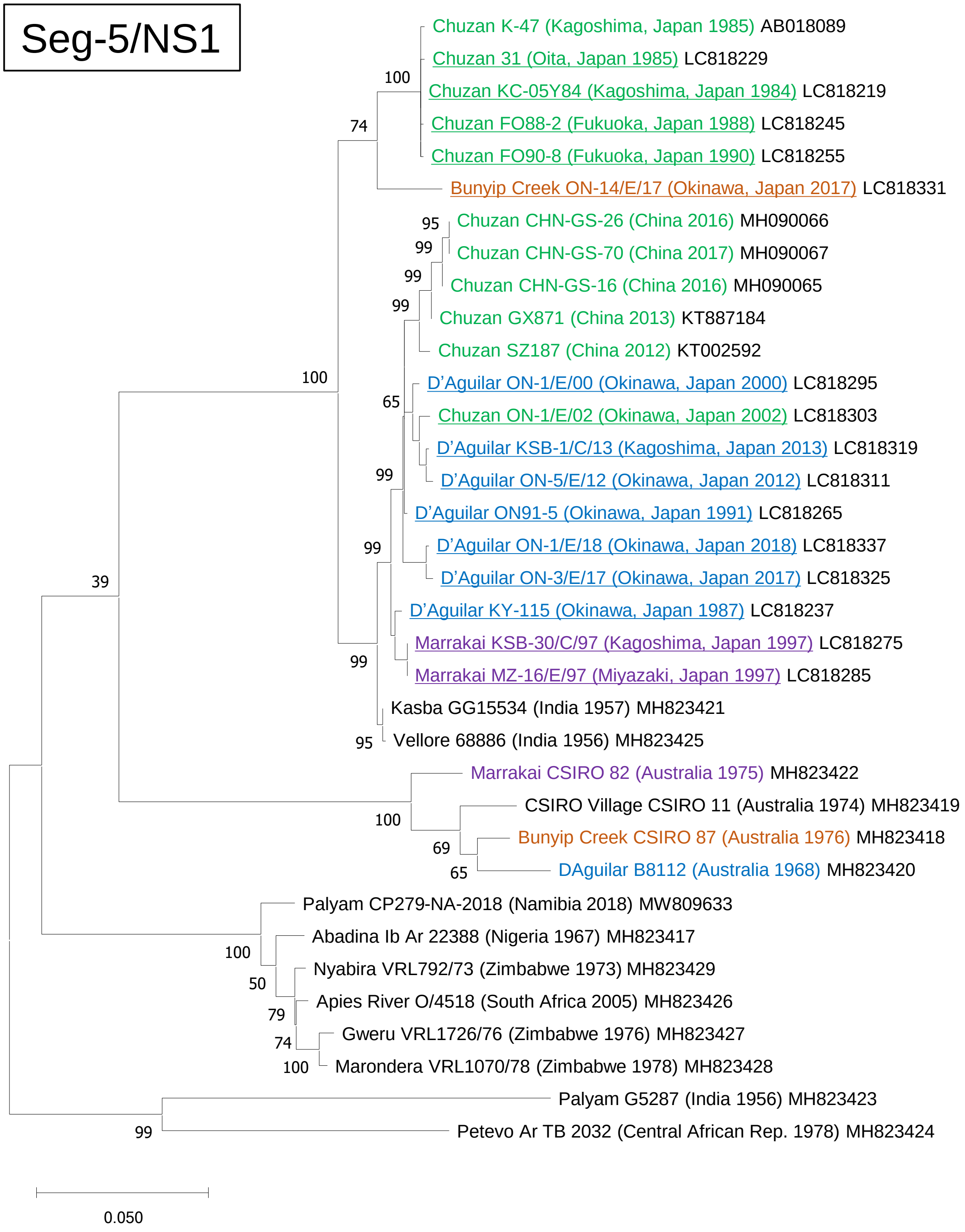

Figure S5. Phylogenetic profile showing the relationships among the Palyam serogroup virus (PALV) strains based on the complete coding region of genome segment 5. The Japanese PALV strains sequenced in this study are underlined. The percentage bootstrap values calculated from 1,000 replications are indicated around the internal nodes. The scale represents 0.05% sequence divergence.

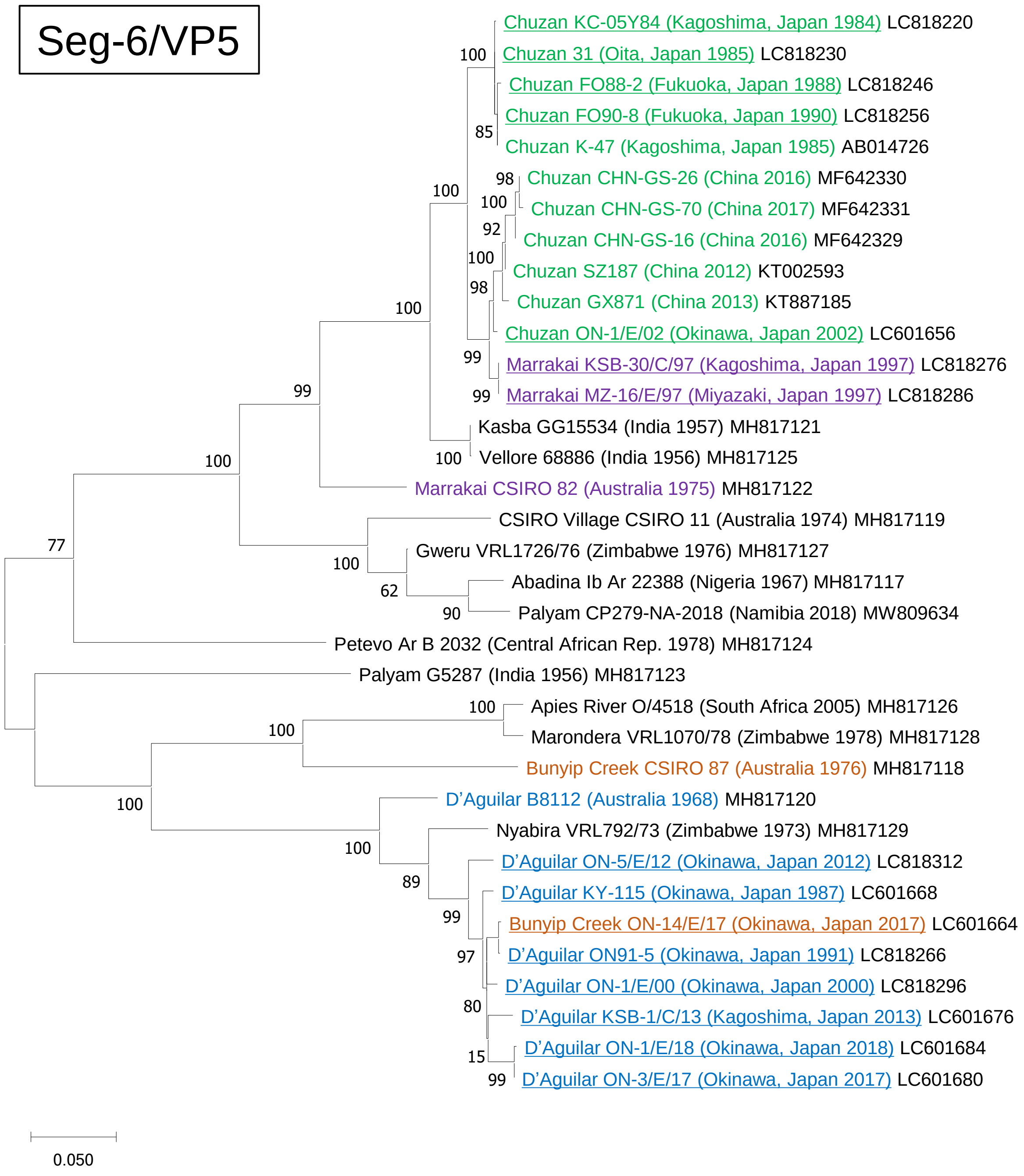

Figure S6. Phylogenetic profile showing the relationships among the Palyam serogroup virus (PALV) strains based on the complete coding region of genome segment 6. The Japanese PALV strains sequenced in this study are underlined. The percentage bootstrap values calculated from 1,000 replications are indicated around the internal nodes. The scale represents 0.05% sequence divergence.

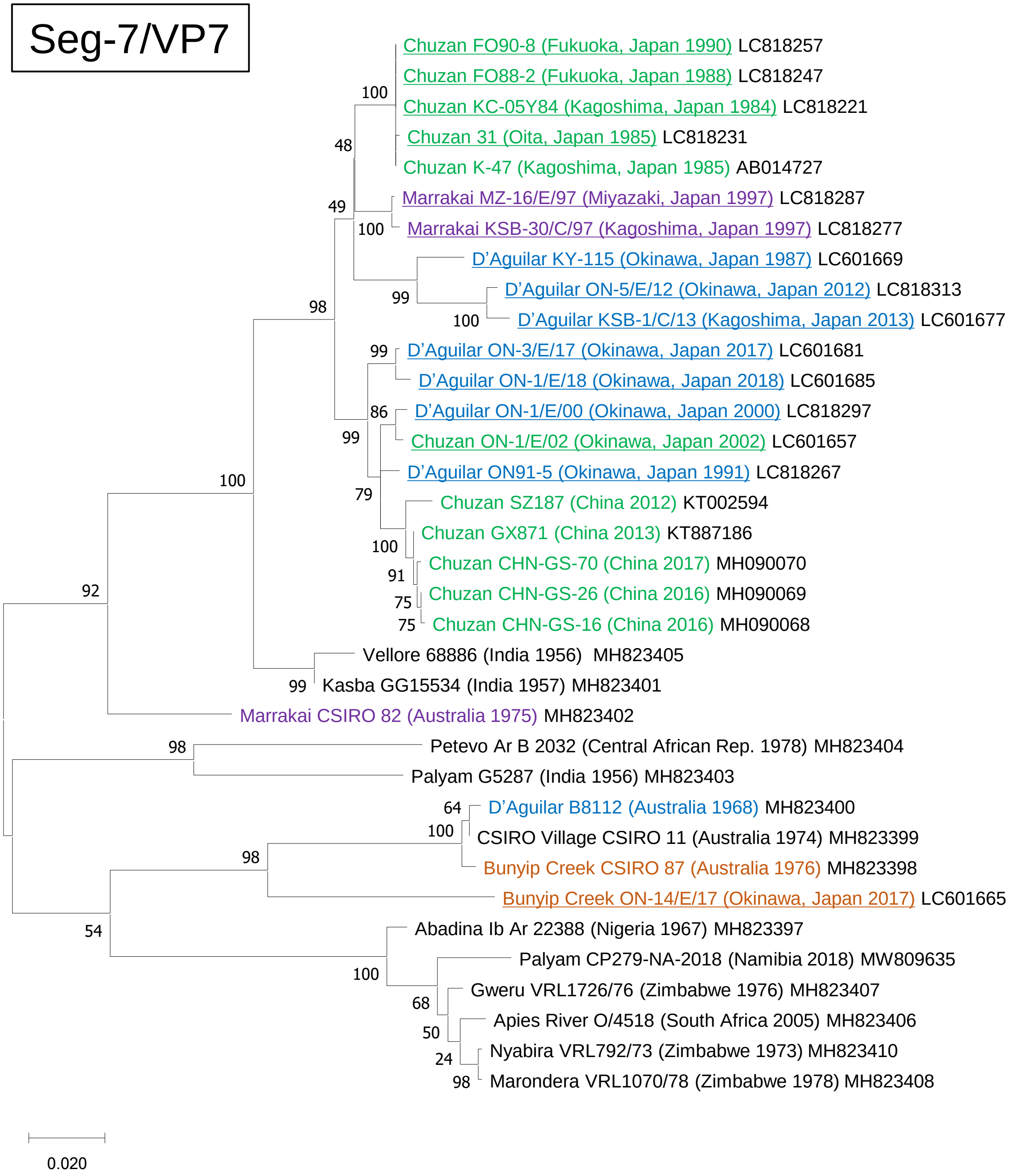

Figure S7. Phylogenetic profile showing the relationships among the Palyam serogroup virus (PALV) strains based on the complete coding region of genome segment 7. The Japanese PALV strains sequenced in this study are underlined. The percentage bootstrap values calculated from 1,000 replications are indicated around the internal nodes. The scale represents 0.02% sequence divergence.

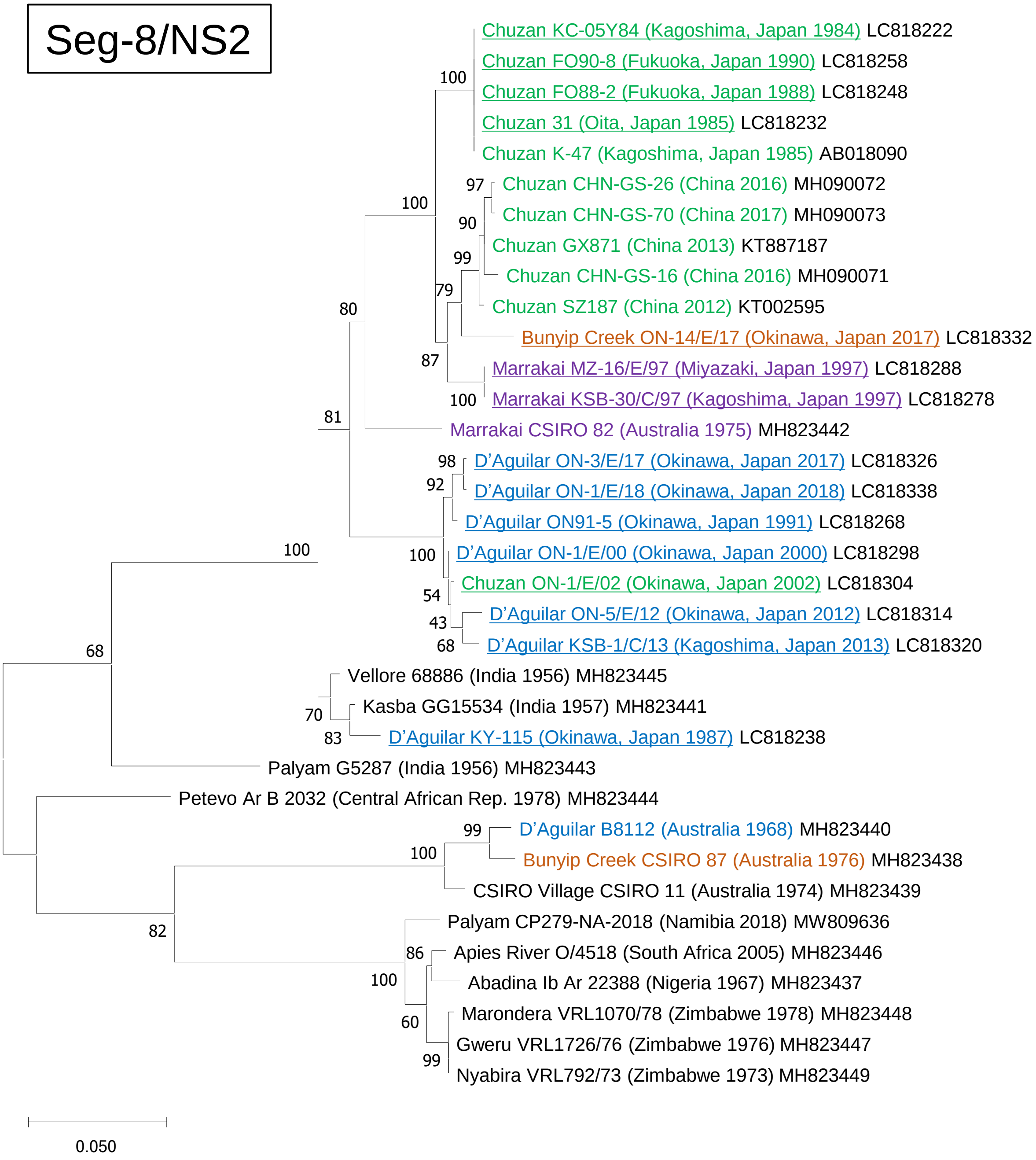

Figure S8. Phylogenetic profile showing the relationships among the Palyam serogroup virus (PALV) strains based on the complete coding region of genome segment 8. The Japanese PALV strains sequenced in this study are underlined. The percentage bootstrap values calculated from 1,000 replications are indicated around the internal nodes. The scale represents 0.05% sequence divergence.

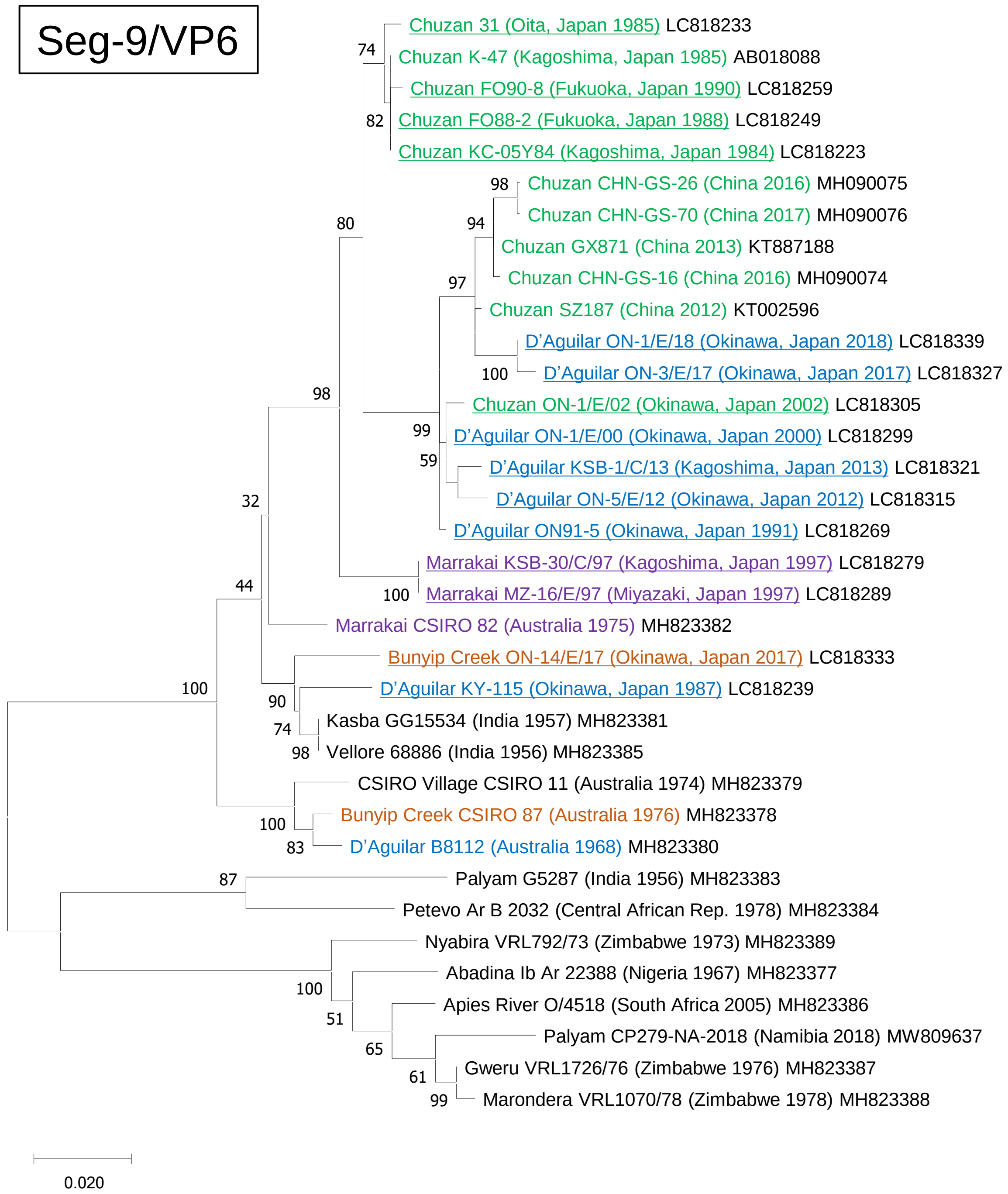

Figure S9. Phylogenetic profile showing the relationships among the Palyam serogroup virus (PALV) strains based on the complete coding region of genome segment 9. The Japanese PALV strains sequenced in this study are underlined. The percentage bootstrap values calculated from 1,000 replications are indicated around the internal nodes. The scale represents 0.02% sequence divergence.

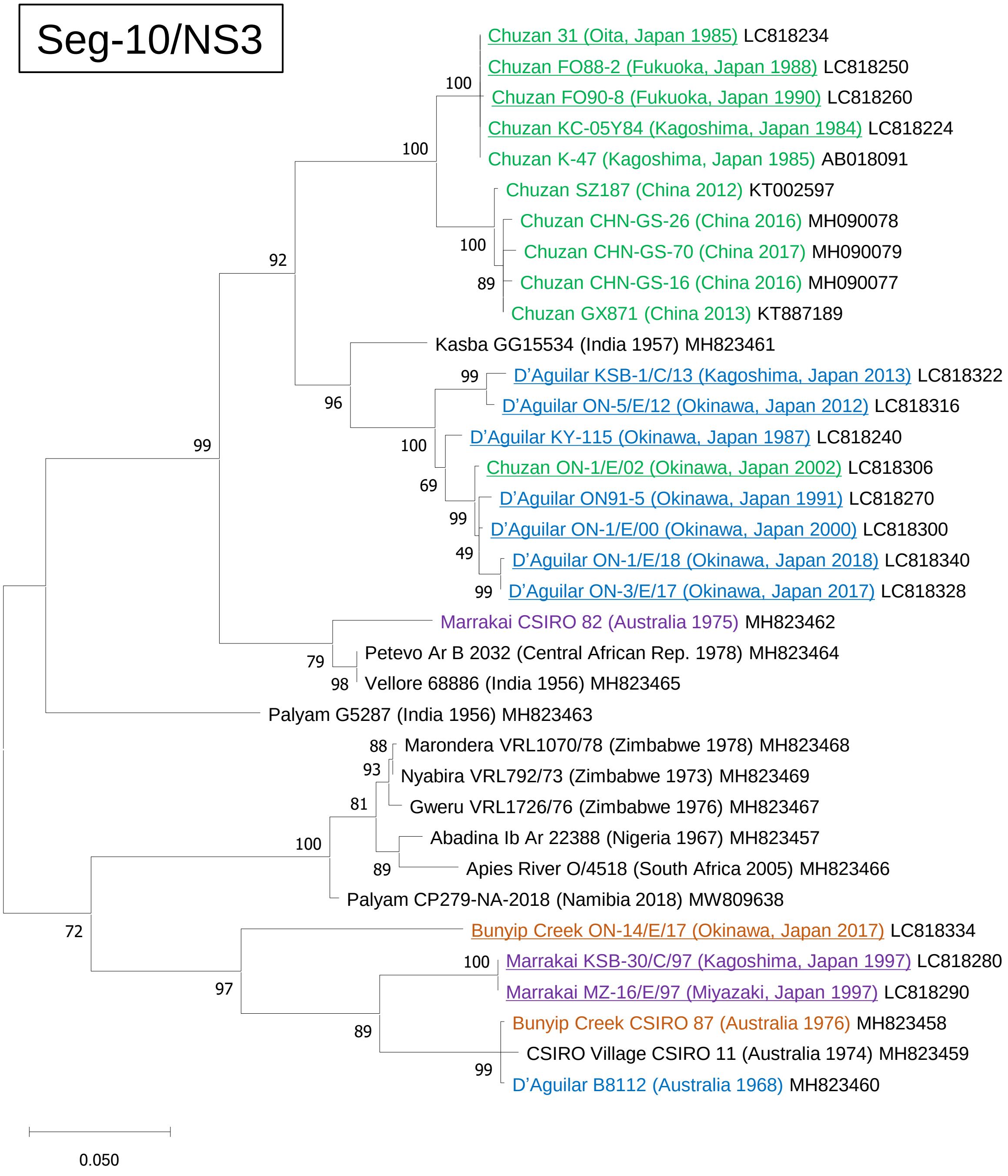

Figure S10. Phylogenetic profile showing the relationships among the Palyam serogroup virus (PALV) strains based on the complete coding region of genome segment 10. The Japanese PALV strains sequenced in this study are underlined. The percentage bootstrap values calculated from 1,000 replications are indicated around the internal nodes. The scale represents 0.05% sequence divergence.

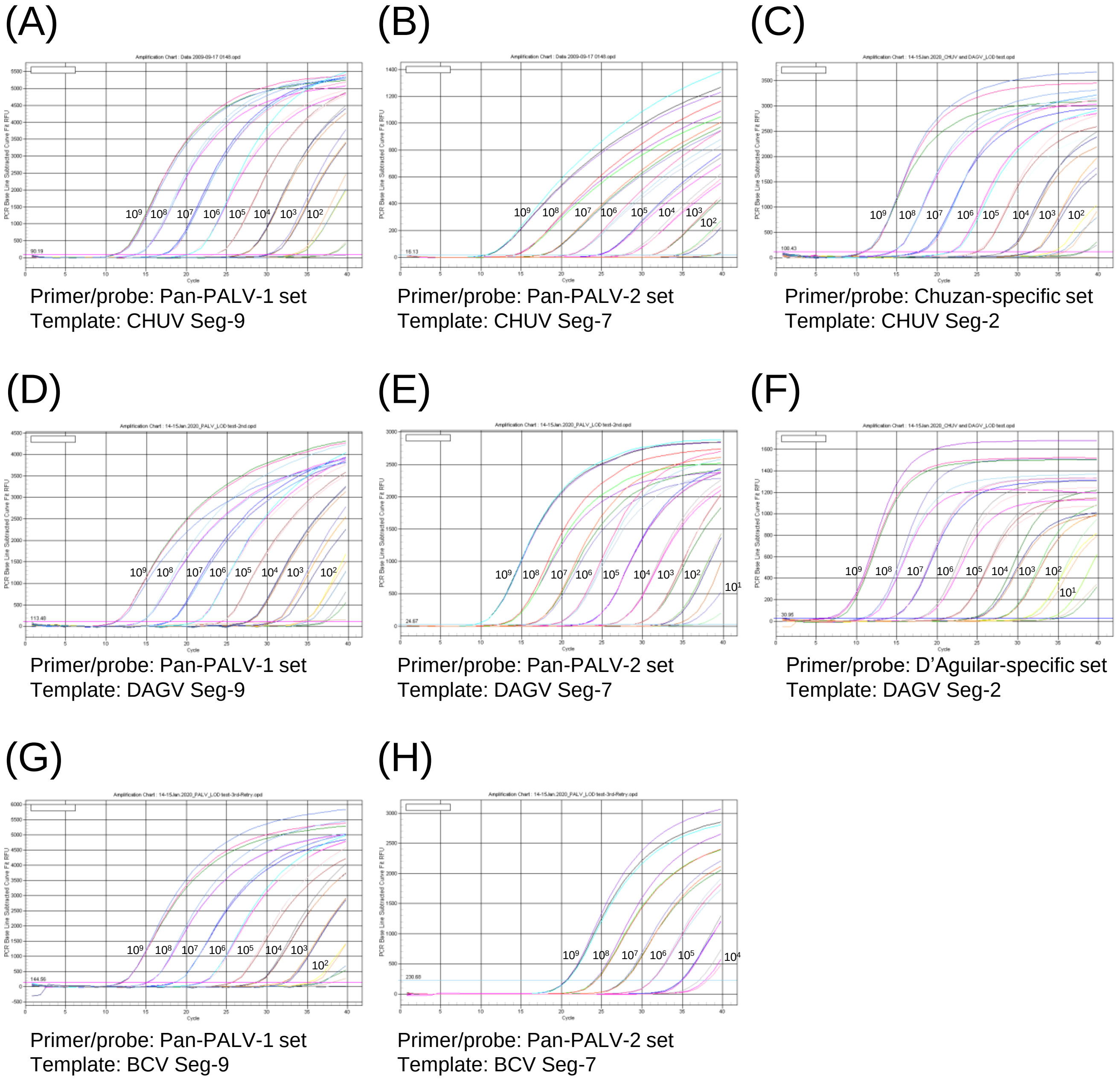

Figure S11. Amplification chart of the real-time RT-PCR assay obtained by testing artificial RNA templates of Chuzan, D'Aguilar and Bunyip Creek viruses. Ten-fold serial dilutions (10^9–10^1 copies/tube) of the templates were subjected to the real-time RT-PCR assay in triplicate. Limits of detection are as follows: (A) 100 copies/tube (Pan-PALV-1 set for CHUV Seg-9 gene), (B) 100 copies/tube (Pan-PALV-2 set for CHUV Seg-7 gene). (C) 100 copies/tube (Chuzan-specific set for CHUV Seg-2 gene), (D) 100 copies/tube (Pan-PALV-1 set for DAGV Seg-9 gene), (E) 10 copies/tube (Pan-PALV-2 set for DAGV Seg-7 gene), (F) 10 copies/tube (D'Aguilar-specific set for DAGV Seg-2 gene), (G) 100 copies/tube (Pan-PALV-1 set for BCV Seg-9 gene), (H) 10,000 copies/tube (Pan-PALV-2 set for BCV Seg-7 gene).
