# Supplementary material for: Genetic Characterization of Palyam Serogroup Viruses Isolated in Japan from 1984 to 2018 and Development of a Real-Time RT-PCR Assay for Broad Detection of Palyam Serogroup Viruses and Specific Detection of Chuzan (Kasba) and D’Aguilar Viruses
Source: Pathogens. 2024 Jun 28;13(7):550. doi: 10.3390/pathogens13070550 (PMC11279806; doi:10.3390/pathogens13070550)
Supplement: Supplementary file 1 [file pathogens-13-00550-s001.zip › Table S1_Shirafuji et al._28May2024.pdf]

Table S1. Percentage sequence identities of Seg-2/VP2 among Japanese strains of PALV at nucleotide (lower triangle) and **amino acid** (upper triangle, shown in **bold**) levels.

	[Chuzan] KC-05Y84	[Chuzan] 31	[Chuzan] FO-88-2	[Chuzan] FO-90-8	[Chuzan] ON-1/E/02	[Marrakai] KSB-30/C/97	[Marrakai] MZ-16/E/97	[Bunyip Creek] ON-14/E/17	[D'Aguilar] KY-115	[D'Aguilar] ON91-5	[D'Aguilar] ON-1/E/00	[D'Aguilar] ON-5/E/12	[D'Aguilar] KSB-1/C/13	[D'Aguilar] ON-3/E/17	[D'Aguilar] ON-1/E/18
[Chuzan] KC-05Y84		**100**	**99.80**	**99.90**	**98.80**	**44.63**	**44.72**	**41.10**	**40.05**	**40.25**	**39.90**	**39.90**	**39.96**	**40.35**	**40.25**
[Chuzan] 31	99.90		**99.80**	**99.90**	**98.80**	**44.63**	**44.72**	**41.10**	**40.05**	**40.25**	**39.90**	**39.90**	**39.96**	**40.35**	**40.25**
[Chuzan] FO-88-2	99.93	99.83		**99.90**	**98.60**	**44.63**	**44.72**	**41.10**	**40.15**	**40.35**	**40.00**	**40.00**	**40.05**	**40.45**	**40.35**
[Chuzan] FO-90-8	99.86	99.00	99.93		**98.70**	**44.53**	**44.63**	**41.00**	**40.15**	**40.35**	**40.00**	**40.00**	**40.05**	**40.45**	**40.35**
[Chuzan] ON-1/E/02	98.13	98.10	98.00	98.07		**45.02**	**45.12**	**41.10**	**40.45**	**40.45**	**40.09**	**40.09**	**40.15**	**40.55**	**40.45**
[Marrakai] KSB-30/C/97	55.13	55.29	55.18	55.29	54.97		**99.90**	**39.94**	**38.21**	**38.31**	**38.50**	**38.34**	**38.50**	**38.40**	**38.31**
[Marrakai] MZ-16/E/97	55.10	55.32	55.21	55.32	55.00	99.96		**40.03**	**38.21**	**38.31**	**38.50**	**38.24**	**38.40**	**38.40**	**38.31**
[Bunyip Creek] ON-14/E/17	53.99	54.02	54.02	53.96	54.07	53.12	53.16		**47.12**	**47.12**	**47.12**	**47.02**	**47.51**	**47.22**	**47.22**
[D'Aguilar] KY-115	52.76	52.83	52.83	52.80	52.70	52.79	52.55	56.49		**98.38**	**98.08**	**98.18**	**98.08**	**97.98**	**97.78**
[D'Aguilar] ON91-5	52.72	52.78	52.62	52.49	52.60	52.73	53.10	56.18	95.56		**99.49**	**97.78**	**97.68**	**99.19**	**98.99**
[D'Aguilar] ON-1/E/00	52.68	52.75	52.58	52.58	52.52	53.23	53.20	56.17	95.23	99.06		**97.47**	**97.37**	**98.89**	**98.68**
[D'Aguilar] ON-5/E/12	52.46	52.64	52.64	52.60	52.77	52.73	52.76	56.39	96.94	94.96	94.69		**98.79**	**97.58**	**97.37**
[D'Aguilar] KSB-1/C/13	52.24	52.42	52.42	52.39	52.26	52.91	52.95	56.96	96.64	94.76	94.49	98.69		**97.47**	**97.27**
[D'Aguilar] ON-3/E/17	52.45	52.52	52.49	52.49	52.57	52.86	52.89	56.30	94.32	98.22	97.68	93.99	93.72		**99.79**
[D'Aguilar] ON-1/E/18	52.45	52.52	52.49	52.49	52.57	52.79	52.82	56.30	94.25	98.15	97.61	93.92	93.65	99.86	
